# Risk of suicide in people living with HIV: A nationwide, retrospective population‐based cohort study in South Korea

**DOI:** 10.1002/jia2.26521

**Published:** 2025-06-05

**Authors:** Tak Kyu Oh, Kyoung‐Ho Song, Eunjeong Heo, Hye Yoon Park, In‐Ae Song

**Affiliations:** ^1^ Department of Anesthesiology and Pain Medicine Seoul National University Bundang Hospital Seongnam South Korea; ^2^ Department of Anesthesiology and Pain Medicine College of Medicine Seoul National University Seoul South Korea; ^3^ Department of Internal Medicine Seoul National University Bundang Hospital Seongnam South Korea; ^4^ Department of Internal Medicine College of Medicine Seoul National University Seoul South Korea; ^5^ Department of Pharmacy Seoul National University Bundang Hospital Seongnam South Korea; ^6^ Department of Psychiatry Seoul National University Bundang Hospital Seongnam South Korea

**Keywords:** HIV, mortality, propensity score, regression analysis, Republic of Korea, suicide

## Abstract

**Introduction:**

There is a paucity of studies that compare suicide‐ and non‐suicide‐related deaths, with strict adjustments for people living with human immunodeficiency virus (HIV; PLWH) and those without HIV. We, therefore, aimed to determine whether the risk of suicide differs between these groups.

**Methods:**

This study included all PLWH diagnosed with HIV in South Korea between 1 January 2017 and 31 December 2017. Individuals who had never been diagnosed with HIV were selected as controls using 1:10 stratified random sampling, considering age and sex. The heterogeneity of covariates between PLWH and controls was decreased by 1:5 propensity score matching. The endpoint of the study was death by suicide, with follow‐up from 1 January 2018 to 31 December 2022. Death that was not ruled as a suicide was categorized as being due to other causes.

**Results:**

After propensity score matching, 22,415 PLWH (mean age 45.9 years; 91% male) and 96,790 controls (mean age 45.8 years; 90.5% male) were included in the final analysis. Within 5 years, 104 (0.5%) of PLWH and 246 (0.3%) of controls died by suicide. Cox regression analysis revealed a 1.84‐fold higher risk of suicide among PLWH compared with controls (hazard ratio [HR], 1.84; 95% confidence interval [CI], 1.46–2.31; *p* < 0.001). Moreover, 836 (3.7%) of 22,415 PLWH and 2882 (3.0%) of 96,790 controls died of other causes within 5 years. Cox regression analysis also revealed a 1.26‐fold increase in the risk of mortality due to other causes among PLWH (HR: 1.26; 95% CI, 1.17–1.36; *p* < 0.001).

**Conclusions:**

This analysis of a South Korean cohort found higher rates of death due to suicide and other causes among people living with and without HIV. The risk of death by suicide was higher than that of other causes among PLWH.

## 1 INTRODUCTION

Since the first reports of acquired immune deficiency syndrome (AIDS) during the 1980s [1], the prevalence of human immunodeficiency virus (HIV)‐related fatalities has increased [2], including those caused by opportunistic infections and acute infection syndrome. In 2020, 1.5 million new cases of HIV acquisitions and 37.7 million persons living with HIV (PLWH) were reported worldwide by the Joint United Nations Programme on HIV [3]. Since the beginning of the coronavirus disease pandemic, 36.3 million individuals have died of AIDS‐related illnesses [3]. Anti‐retroviral therapy (ART) has significantly increased the life expectancy of persons who are HIV positive. Nonetheless, HIV is presently one of the biggest global health issues because ART cannot cure AIDS [4].

The global impact of suicide is significant, accounting for 13% of all deaths in 2019 [5]. The prevalence of suicide in South Korea is a pressing concern, with a rate of 246 per 100,000 individuals in 2019, the highest among countries in the Organization for Economic Cooperation and Development [6]. The prevalence of suicide between 2011 and 2016 in South Korea was significant compared with the total number of deaths (84,934 [5.26%] of 1,615,288) [7]. An important health issue for PLWH is psychiatric illnesses such as depression [8], which is the most prevalent neuropsychiatric complication among PLWH. The prevalence of depression in PLWH ranges from 22% to 71% [9], and social discrimination and stigma are often cited as the causes [10]. The social stigma associated with HIV elevates suicidal ideation among PLWH [11], and a lack of social support is a risk factor for suicide in PLWH [12]. A recent meta‐analysis found a 100‐fold increased risk of suicide mortality among PLWH compared with the general population [13]. However, there is a paucity of studies that compare suicide‐ and non‐suicide‐related deaths between PLWH and those without HIV. This is a significant health concern in South Korea, where the mortality rate due to suicide (25.4 per 100,000 individuals) was the fourth highest in the world and highest among high‐income countries in the Organization for Economic Cooperation and Development in 2019 [14]. Therefore, we aimed to determine whether the risk of death by suicide differs between PLWH and those without HIV.

## 2 METHODS

### 2.1 Study design

This population‐based cohort study complied with the guidelines of Strengthening the Reporting of Observational Studies in Epidemiology (STROBE) [15].

### 2.2 Ethics

The Institutional Review Board at Seoul National University Bundang Hospital approved this study (Approval ID: X‐2306‐837‐902; clearance date 2023‐06‐20) and waived the need for written, informed consent as we retrospectively analysed innominate data. The National Health Insurance Service (NHIS) approved the study protocol (Approval ID: NHIS‐2024‐1‐021) and authorized access to their database.

### 2.3 NHIS database

The NHIS is the sole public health insurance provider in South Korea. It collects and maintains a vast amount of data on orders for prescription drugs, healthcare operations and disease diagnoses. The data are arranged and classified using International Classification of Diseases, Tenth Revision (ICD‐10) codes. All Koreans living in South Korea and foreign nationals who have resided there for > 6 months must enrol in the NHIS programme. The government subsidizes treatments and tests paid by NHIS enrolees, depending on the severity of their ailments. Although > 95% of healthcare providers are private, the government regulates prescriptions, treatments and fees. Consequently, no information was available regarding the possibility of missing prescriptions or diagnoses by doctors. In addition to primary diagnoses, all ICD‐10 diagnoses were considered. Statistics on socio‐economic factors and overall mortality rates are also included in the NHIS database [16].

### 2.4 PLWH in South Korea

South Korean law categorizes HIV as a Group 3 infectious disease, and the Korea Disease Control and Prevention Agency (KCDPA) is responsible for its management. All healthcare facilities can screen for HIV positivity using enzyme‐linked immunosorbent assays (ELISA). Positive ELISA findings are then sent to the KCDPA and any of the 17 Institutes of Health nationwide to rule out or confirm HIV acquisition by western blotting. As soon as a public health centre is notified of positive HIV results, physicians who ordered the tests are obligated to inform their patients. The ICD‐10 codes B20–B24 listed in the NHIS database must also be associated with a physician.

PLWH are entitled to state reimbursement for all related medical and treatment expenses as HIV is legally categorized as a Group 3 infectious disease. One aspect of this approach is the prescription of ART drugs.

### 2.5 Study population (PWLH and controls)

This study included all PLWH diagnosed with HIV in South Korea between 1 January 2017 and 31 December 2017. Based on earlier research in a Japanese cohort study [17], four ICD codes were used to identify PLWH: non‐specific HIV disease (B24), malignant neoplasms (B21), other specified diseases (B22), other disorders (B23), and HIV diseases resulting in infectious and parasitic diseases (B20). The number of PLWH in 2017 is a cumulative statistic that included persons who were diagnosed with HIV for the first time during and before 2017.

After extracting data of all PLWH in 2017 in South Korea using 1:10 stratified random sampling, considering age and sex, we requested the extraction of data for individuals who had never been diagnosed with HIV (ICD‐10 codes B20–B24) and assigned them as controls. We followed the deaths of PLWH and controls between 1 January 2018 and 31 December 2022, and excluded those who died in 2017.

### 2.6 Study endpoint

The endpoint of the study was death by suicide, with follow‐up between 1 January 2018 and 31 December 2022. We applied the ICD‐10 codes X60–X84 to extract data on deaths due to suicide, as described in a previous study [18]. In South Korea, all accurate causes of death are registered by physicians and archived in the Statistics Korea Database. Statistics Korea is a central government agency that provides services related to the planning and coordination of national statistics. In addition to death by suicide, we also categorized all other deaths due to non‐suicidal causes as “other”. Death by suicide is categorized as unnatural in South Korea; thus, physicians must report such events to law enforcement authorities. When the circumstances of suicide are ambiguous, the law mandates an autopsy under the supervision of the prosecutor to ascertain the cause of death. Consequently, underreported suicides in South Korea are unlikely.

### 2.7 Collected covariates

Patient age and sex were also assessed. Covariates for socio‐economic status (SES) included household income and work status (including self‐employment). Five household income levels were stratified, one of which had a medical aid programme with four quartiles. The government classifies those living in poverty and who cannot afford insurance as groups requiring medical aid.

Comorbidities were evaluated using the Charlson Comorbidity Index (CCI) and underlying disability. CCI scores at the time of hospital admission were calculated using ICD‐10 codes from the NHIS database (Table S1). All disabilities in South Korea must be reported in the NHIS database before patients are eligible for benefits provided by social welfare programmes. Every recognized disability must be legally identified by a medical professional who evaluates difficulties with activities of daily living. Table S2 presents a comprehensive classification of the disabilities. Patients were assigned to one of the six categories based on the severity of symptoms. Grades 1–3 and 4–6 were classified as “severe” and “mild to moderate,” respectively.

Underlying psychiatric morbidities were collected as covariates because they could affect the risk of death due to suicide. Psychiatric morbidities included anxiety (F40, F41), bipolar (F31), manic (F30) and substance abuse (F10–F19) disorders, as well as depression (F32, F33, F34.1), previous suicide attempt/self‐harm (X60–X84, Y87.0) and schizophrenia (F20).

### 2.8 Statistical analysis

Clinicopathological differences between PLWH and controls are presented as means with standard deviations (SD) for continuous variables and as numbers with percentages (%) for categorical variables. Because propensity score (PS) matching can mitigate bias in observational studies [19], we performed PS matching to decrease the heterogeneity of covariates between PLWH and controls. In particular, PS matching was applied without replacement at a 1:5 ratio and with a caliper width of 0.25 using the nearest‐neighbour approach. Thereafter, the balance between the groups was assessed using the absolute value of the standardized mean difference (ASD). If the ASD was < 0.1, PS matching was considered appropriate.

In the PS‐matched cohort, we performed Cox regression analyses to examine whether the risk of death due to suicide and other causes differed between PLWH and controls. In this time‐to‐event analysis, we defined an event as death by suicide or other causes that occurred on or after 1 January 2018, and defined time frames as the period from 1 January 2018 until the date of the event. In addition, the cumulative incidence of suicide and other deaths in PLWH and controls were determined and assessed using competing risk analyses. Next, we performed a sensitivity analysis using multivariable Cox regression modelling to examine whether the results obtained in the PS‐matched cohort were similar to those of the entire cohort. All covariates were included in the multivariable‐adjusted model. We fitted another multivariable Cox regression model to examine whether the main results differed among PLWH on ART. Finally, we performed subgroup analyses according to sex, underlying psychiatric morbidity, patient age, CCI, underlying cancer, disability, employment status and household income. We ensured that the fundamental assumptions of the Cox proportional hazards models were satisfied using log‐log plots. All data are presented as hazard ratio (HR)s with 95% confidence interval (CI)s. The multivariable model did not reveal any multicollinearity between the variables at a variance inflation factor of 2. All data were statistically analysed using R version 4.0.3 (R Foundation for Statistical Computing, Vienna, Austria) with a significance threshold of *p* < 0.05.

## 3 RESULTS

### 3.1 Study population

**Figure 1:**
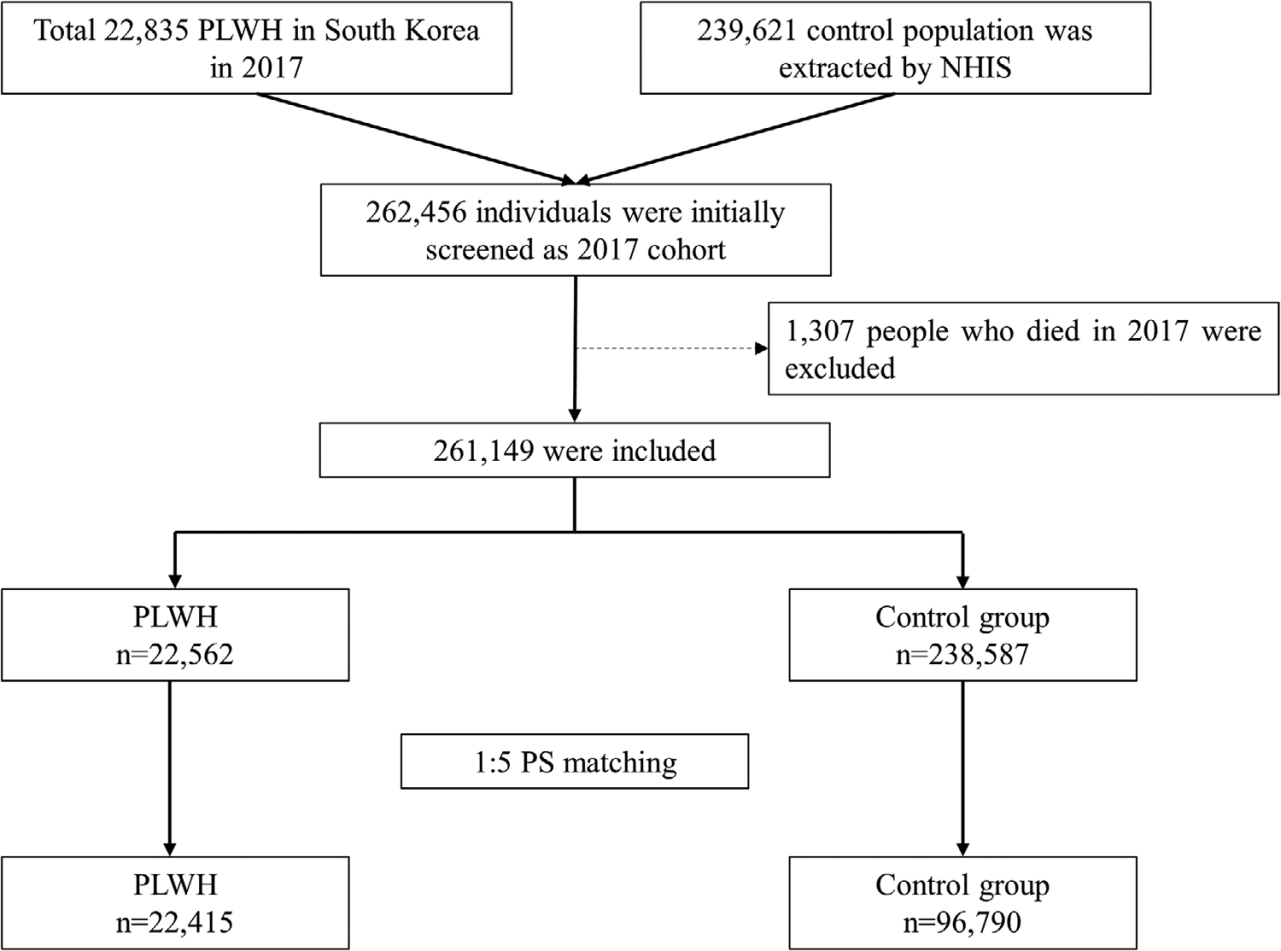
Flowchart of patients through the study. NHIS, national health insurance service; PLWH, people living with human immunodeficiency virus; PS, propensity score.

Figure 1 shows the flowchart of the patient selection process. We extracted the data of 22,835 PLWH in South Korea in 2017 and 239,621 controls from the NHIS database. We excluded 1307 individuals who died in 2017, which left 261,149 patients. After 1:5 PS matching, 22,415 PLWH and 96,790 controls were included in the final analysis.

Table 1 presents the clinicopathological characteristics of the PLWH and controls before and after PS matching. All ASDs were < 0.1 in the PS‐matched cohort, suggesting that the two groups were adequately balanced. Figure S1 shows that the distribution became similar after PS matching.

**Table 1:** Clinicopathological characteristics of PLWH and controls before and after PS matching

	Entire cohort (*n* = 261,149)			PS‐matched cohort (*n* = 119,205)		
Variable	PLWH *n* = 22,562	Control *n* = 238,587	ASD	PLWH *n* = 22,415	Control *n* = 96,790	ASD
Age (years)	46.0 (13.8)	46.7 (14.3)	0.057	45.9 (13.8)	45.8 (14.3)	0.042
Male	20,539 (91.0)	212,324 (89.0)	0.071	20,398 (91.0)	87,609 (90.5)	0.028
Employed	11,532 (51.1)	161,591 (67.7)	0.332	11,532 (51.4)	56,722 (58.6)	0.009
Household income						
Medical aid programme group	3481 (15.4)	5708 (2.4)		3336 (14.9)	5496 (5.7)	
Q1	4097 (18.2)	39,930 (16.7)	0.052	4095 (18.3)	18,854 (19.5)	0.011
Q2	5258 (23.3)	50,374 (21.1)	0.139	5258 (23.5)	23,963 (24.8)	0.003
Q3	4766 (21.1)	63,936 (26.8)	0.246	4766 (21.3)	23,301 (24.1)	0.015
Q4	4777 (21.2)	74,526 (31.2)	0.361	4777 (21.3)	24,157 (25.0)	0.007
Unknown	183 (0.8)	4113 (1.7)	0.102	183 (0.8)	1019 (1.1)	0.010
Underlying disability						
Mild to moderate	958 (4.2)	8689 (3.6)	0.030	951 (4.2)	3525 (3.6)	0.019
Severe	632 (2.8)	5514 (2.3)	0.029	630 (2.8)	2420 (2.5)	0.038
CCI	1.3 (1.7)	0.9 (1.5)	0.269	1.3 (1.7)	1.2 (1.7)	0.042
Myocardial infarction	237 (1.1)	1665 (0.7)	0.035	234 (1.0)	839 (0.9)	0.004
Congestive heart failure	623 (2.8)	4614 (1.9)	0.051	618 (2.8)	2197 (2.3)	0.013
Peripheral vascular disease	1275 (5.7)	12,508 (5.2)	0.018	1265 (5.6)	5017 (5.2)	0.026
Cerebrovascular disease	914 (4.1)	8257 (3.5)	0.030	908 (4.1)	3484 (3.6)	0.026
Dementia	438 (1.9)	3748 (1.6)	0.027	429 (1.9)	1580 (1.6)	0.023
Chronic pulmonary disease	6220 (27.6)	42,147 (17.7)	0.222	6121 (27.3)	23,246 (24.0)	<0.001
Rheumatic disease	550 (2.4)	4348 (1.8)	0.040	539 (2.4)	2112 (2.2)	0.009
Peptic ulcer disease	3451 (15.3)	30,921 (13.0)	0.065	3430 (15.3)	13,863 (14.3)	0.018
Mild liver disease	6657 (29.5)	33,364 (14.0)	0.340	6534 (29.2)	24,007 (24.8)	0.015
DM without chronic complication	3618 (16.0)	25,368 (10.6)	0.147	3545 (15.8)	13,589 (14.0)	0.029
DM with chronic complication	945 (4.2)	7702 (3.2)	0.048	938 (4.2)	3628 (3.7)	0.028
Hemiplegia or paraplegia	179 (0.8)	952 (0.4)	0.044	179 (0.8)	553 (0.6)	0.015
Renal disease	362 (1.6)	2221 (0.9)	0.054	361 (1.6)	1261 (1.3)	0.016
Cancer	1240 (5.5)	8043 (3.4)	0.093	1226 (5.5)	4493 (4.6)	0.014
Moderate or severe liver disease	45 (0.2)	399 (0.2)	0.007	45 (0.2)	156 (0.2)	0.007
Metastatic solid tumour	93 (0.4)	660 (0.3)	0.021	90 (0.4)	344 (0.4)	0.009
Underlying psychiatric morbidities						
Anxiety disorder	2988 (13.2)	21,347 (8.9)	0.127	2950 (13.2)	10,836 (11.2)	0.027
Bipolar disorder	651 (2.9)	2655 (1.1)	0.106	628 (2.8)	1751 (1.8)	0.012
Depression	2933 (13.0)	13,976 (5.9)	0.212	2850 (12.7)	9060 (9.4)	0.017
Manic disorder	25 (0.1)	174 (0.1)	0.011	25 (0.1)	87 (0.1)	0.013
Previous suicide attempt/self‐harm	6 (0.0)	0 (0.0)	<0.001	0 (0.0)	0 (0.0)	<0.001
Schizophrenia	1816 (0.8)	257 (1.1)	0.036	254 (1.1)	922 (1.0)	0.016
Substance use disorder	349 (1.5)	2318 (1.0)	0.047	345 (1.5)	1108 (1.1)	0.025

Abbreviations: ASD, absolute value of standardized mean difference; CCI, Charlson comorbidity index; DM, diabetes mellitus; HIV, human immunodeficiency virus; PLWH, people living with human immunodeficiency virus.

### 3.2 Analysis of the PS‐matched cohort

Table 2 shows the results of the survival analyses before and after PS matching. After PS matching, 0.5% (104/22,415) of PLWH and 0.3% (246/96,790) of controls died within 5 years due to suicide. The Cox regression analysis revealed a 1.84‐fold (HR: 1.84, 95% CI: 1.46, 2.31; *p* < 0.001) higher risk of suicide among PLWH compared with controls. Moreover, 3.7% (836/22,415) of PLWH and 3.0% (2882/96,790) of controls died within 5 years due to other causes. Cox regression analysis revealed a 1.26‐fold increase in the risk of mortality due to other causes among PLWH (HR, 1.26; 95% CI, 1.17–1.36; *p* < 0.001).

**Table 2:** Survival analyses before and after PSM

Variable	Event *n* (%)	HR (95% CI)	*p*
Before PSM			
Death due to suicide			
Control	515 of 238,587 (0.2)	1	
PLWH	104 of 22,562 (0.5)	2.15 (1.74, 2.66)	< 0.001
Death due to other causes			
Control	6368 of 238,587 (2.7)	1	
PLWH	843 of 22,562 (3.7)	1.41 (1.31, 1.52)	< 0.001
After PS matching			
Death due to suicide			
Control	246 of 96,790 (0.3)	1	
PLWH	104 of 22,415 (0.5)	1.84 (1.46, 2.31)	< 0.001
Death due to other causes			
Control	2882 of 96,790 (3.0)	1	
PLWH	836 of 22,415 (3.7)	1.26 (1.17, 1.36)	< 0.001

Abbreviations: HR, hazard ratio; PLWH, people living with human immunodeficiency virus; PS, propensity score.

**Figure 2:**
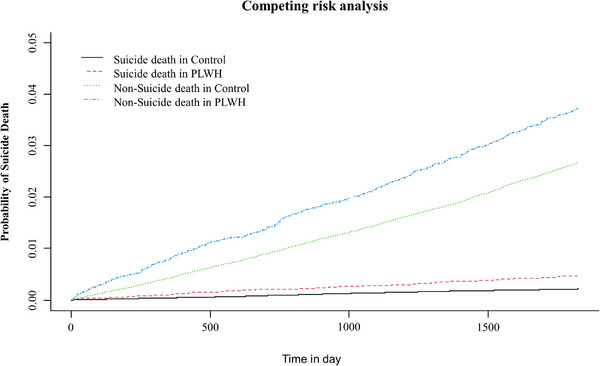
Cumulative incidence of death due to suicide and other causes among PLWH and controls in PSM cohort. PLWH, people living with human immunodeficiency virus; PSM, propensity score matching.

Figure 2 shows the cumulative incidence function of death due to suicide and other causes in PLWH and controls in the PSM cohort. The cumulative incidence of death due to suicide and other causes was higher among PLWH than the controls (*p* < 0.001).

### 3.3 Sensitivity and subgroup analyses of entire cohort

Table 3 shows the results of the multivariable Cox regression model for death due to suicide in the entire cohort. Models 1 and 2 show that the risk of death due to suicide was 1.7‐fold higher among PLWH than controls (HR, 1.70; 95% CI, 1.36–2.12; *p* < 0.001) and 1.67‐fold higher among PLWH receiving ART than controls (HR, 1.67; 95% CI, 1.33–2.08; *p* < 0.001).

**Table 3:** Multivariable Cox regression model of death due to suicide in entire cohort

Variable	HR (95% CI)	*p*
PLWH (*n* = 22,562) (vs. control; *n* = 238,587), model 1	1.70 (1.36, 2.12)	<0.001
^a^ART user in PLWH (*n* = 22,268) (vs. control; *n* = 238,587), model 2	1.67 (1.33, 2.08)	<0.001
Other covariates in model 1		
Age	1.01 (1.01, 1.02)	0.001
Male	2.79 (1.92, 4.04)	<0.001
Employed	0.65 (0.55, 0.77)	<0.001
Household income		
Medical aid programme group	0.71 (0.49, 1.03)	0.0698
Q1	1	
Q2	0.94 (0.74, 1.18)	0.589
Q3	0.69 (0.54, 0.88)	0.002
Q4	0.56 (0.44, 0.71)	<0.001
Unknown	0.79 (0.39, 1.62)	0.524
Underlying disability		
Mild to moderate	0.94 (0.65, 1.38)	0.763
Severe	0.96 (0.62, 1.50)	0.865
Myocardial infarction	1.15 (0.57, 2.298)	0.694
Congestive heart failure	1.60 (1.06, 2.43)	0.026
Peripheral vascular disease	1.08 (0.79, 1.48)	0.636
Cerebrovascular disease	0.91 (0.62, 1.35)	0.644
Dementia	0.95 (0.58, 1.56)	0.838
Chronic pulmonary disease	1.05 (0.86, 1.28)	0.614
Rheumatic disease	0.62 (0.32, 1.21)	0.158
Peptic ulcer disease	1.08 (0.87, 1.35)	0.499
Mild liver disease	0.96 (0.78, 1.20)	0.744
DM without chronic complication	0.97 (0.75, 1.26)	0.827
DM with chronic complication	1.08 (0.73, 1.61)	0.706
Hemiplegia or paraplegia	1.71 (0.80, 3.67)	0.167
Renal disease	0.84 (0.41, 1.75)	0.645
Cancer	0.92 (0.60, 1.39)	0.686
Moderate or severe liver disease	0.00 (0.00‐)	0.867
Metastatic solid tumour	2.14 (0.75, 6.13)	0.155
Underlying psychiatric morbidities		
Anxiety disorder	1.42 (1.12, 1.81)	0.004
Bipolar disorder	2.07 (1.40, 3.05)	<0.001
Depression	1.89 (1.46, 2.45)	<0.001
Manic disorder	1.63 (0.51, 5.19)	0.413
Previous suicidal and self‐harm attempt	0.00 (0.00‐)	0.984
Schizophrenia	1.57 (0.96, 2.57)	0.074
Substance use disorder	1.86 (1.23, 2.80)	0.003

Abbreviations: ART, anti‐retroviral therapy; CI, confidence interval; DM, diabetes mellitus; HIV, human immunodeficiency virus; HR, hazard ratio; PLWH, people living with human immunodeficiency virus.

^a^ PLWH receiving ART were included in a separate multivariable model to avoid multi‐collinearity with PLWH.

Table 4 shows the results of the subgroup analyses. The risk of suicide death was higher in PLWH than controls in the male sex group (HR: 1.75, 95% CI: 1.40, 2.19; *p* < 0.001) but not in the female sex group (*p* = 0.526). The risk of suicide death was higher in PLWH than in controls among those aged ≥ 61 (HR, 1.88, 95% CI, 1.18, 3.01; *p* = 0.008) and 41–60 years (HR, 1.67, 95% CI, 1.19, 2.33; *p* = 0.003). The risk of death due to suicide was higher among PLWH with CCI ≥ 2 (HR, 1.99; 95% CI, 1.39–2.85; *p* < 0.001), CCI, 0–1 (HR, 1.74; 95% CI, 1.32–2.30; *p* < 0.001) and with underlying cancer (HR, 3.22; 95% CI, 1.43–7.23; *p* < 0.001) than controls.

**Table 4:** Subgroup analyses for suicide death

Variable	HR (95% CI)	*p*‐value
Male sex (*n* = 232,863)		
PLWH (vs. control)	1.75 (1.40, 2.19)	<0.001
Female sex (*n* = 28,286)		
PLWH (vs. control)	0.62 (0.14, 2.69)	0.526
Underlying psychiatric morbidity group (*n* = 34,839)		
PLWH (vs. control)	1.57 (1.09, 2.26)	0.016
Underlying no psychiatric morbidity group (*n* = 226,310)		
PLWH (vs. control)	1.73 (1.31, 2.29)	<0.001
Age: 0–20 (*n* = 1670)		
PLWH (vs. control)	0.02 (0.00‐)	0.803
Age: 21–40 (*n* = 90,782)		
PLWH (vs. control)	1.43 (0.96, 2.12)	0.077
Age: 41–60 (*n* = 123,634)		
PLWH (vs. control)	1.67 (1.19, 2.33)	0.003
Age: ≥61 (*n* = 45,063)		
PLWH (vs. control)	1.88 (1.18, 3.01)	0.008
CCI ≥2 (*n* = 55,967)		
PLWH (vs. control)	1.99 (1.39, 2.85)	<0.001
CCI: 0–1 (*n* = 205,182)		
PLWH (vs. control)	1.74 (1.32, 2.30)	<0.001
Underlying cancer group (*n* = 9315)		
PLWH (vs. control)	3.22 (1.43, 7.23)	0.005
No underlying cancer group (*n* = 251,834)		
PLWH (vs. control)	1.69 (1.35, 2.12)	<0.001
Underlying no disability group (*n* = 245,356)		
PLWH (vs. control)	1.83 (1.46, 2.29)	<0.001
Underlying mild to moderate disability group (*n* = 9647)		
PLWH (vs. control)	1.96 (0.66, 5.81)	0.222
Underlying severe disability group (*n* = 3631)		
PLWH (vs. control)	0.86 (0.66, 1.12)	0.262
Having a job group (*n* = 173,123)		
PLWH (vs. control)	2.02 (1.47, 2.78)	<0.001
Unemployment group (*n* = 88,026)		
PLWH (vs. control)	1.57 (1.18, 2.11)	0.002
Household income level: MAP group (*n* = 9189)		
PLWH (vs. control)	1.50 (0.82, 2.74)	0.188
Household income level: Q1 group (*n* = 44,027)		
PLWH (vs. control)	1.22 (0.72, 2.09)	0.460
Household income level: Q2 group (*n* = 55,632)		
PLWH (vs. control)	1.41 (0.89, 2.23)	0.149
Household income level: Q3 group (*n* = 68,702)		
PLWH (vs. control)	1.80 (1.09, 2.99)	0.022
Household income level: Q4 group (*n* = 79,303)		
PLWH (vs. control)	2.73 (1.71, 4.37)	<0.001

Abbreviations: CI, confidence interval; HR, hazard ratio; MAP, medical aid programme; PLWH, people living with human immunodeficiency virus.

## 4 DISCUSSION

This population‐based cohort study in South Korea revealed higher HRs and a higher risk of death due to suicide and other causes among PLWH compared with controls. Multivariable Cox regression modelling showed that these associations were significant in both the PS‐matched and entire cohorts. We compared PLWH who were integrated with the general population to the remaining general population (controls), as most PLWH were receiving ART. This study is significant because it clearly shows an elevated risk of suicide among PLWH. We also emphasized the issue of deaths due to other causes.

In a previous systematic review and meta‐regression of 43 studies [20], the rates of suicidal ideation, attempted suicide and death by suicide were 22.3%, 9.6% and 1.7%, respectively. In this study, suicide death rates among PLWH in South Korea were 0.5% for 5 years (2018–2022). A recent meta‐analysis revealed a 100‐fold higher risk of suicide‐related mortality among PLWH than the general population [13]. Nevertheless, many studies that have examined the risk of suicide in the general population and PLWH have not thoroughly considered distinct groups [13, 20].

In subgroup analysis, the risk of death by suicide was higher in men than in women. The likelihood of suicide is higher among sexual minorities such as men who have sex with men and others who are disproportionately affected by HIV, such as transgender women [21]. Moreover, sexual minority status itself is a significant factor in increased suicide risk [22]. Men comprised 91.6% of PLWH in South Korea between 2008 and 2015 [23]; the 2017 cohort of our study also revealed a high percentage of men among PLWH (91%). The high rate of HIV acquisition among men is linked to sexual behaviour among sexual minorities, which is the primary route of HIV transmission [24]. The elevated suicide rate among male sexual minorities among PLWH can be attributed to negative societal perceptions associated with both their sexual orientation and HIV status [24]. Hence, it is crucial to consider these factors when addressing suicide prevention among PLWH and developing relevant policies.

Furthermore, the risk of suicide mortality has notably increased among PLWH aged > 40 years. The increased risk of suicide with advancing age poses a significant public health concern on a global scale [25]. In South Korea, the high suicide rate among older individuals is an important social issue [26]. The prevalence of PLWH aged ≥ 50 years is also increasing worldwide, highlighting the need for specific health policies [27]. Thus, our finding on the elevated risk of suicide among PLWH aged > 40 years should be considered when developing strategies to prevent suicide.

This study also revealed a higher risk of suicide among PLWH with a CCI ≥ 2 or underlying cancer. In addition, the HRs for suicide mortality were higher among PLWH without diagnosed psychiatric comorbidities than among those with such conditions. These results suggest that among PLWH, physical comorbidities such as cancer may contribute to increased suicide mortality risk. However, the role of psychiatric comorbidities remains complex and may be influenced by differential access to mental health services and diagnosis. The absence of data on undiagnosed or untreated psychiatric conditions warrants a cautious interpretation of these patterns. Supporting this, a recent case‐control study found that chronic diseases—especially comorbidities—are closely associated with suicide in South Korea [28]. Cancer, in particular, is a significant public health issue that has been linked to increased psychosocial distress and suicide risk [29]. PLWH are known to be at elevated risk for a variety of physical comorbidities, including diabetes, cancer and cardiovascular, liver, and bone diseases [30]. Taken together, our findings indicate that PLWH with multiple comorbidities may be at heightened risk for suicide and should be considered a key population in suicide prevention policy and intervention efforts.

Relationships between SES and the risk of suicide death among PLWH should be carefully interpreted. We found that PLWH who are employed or have a high household income were at increased risk of suicide. While a low SES increases the risk of suicide in South Korea [31], this trend did not apply to PLWH. This difference may be attributed to the full coverage provided by the government for all medical visits, laboratory tests, medications and treatments for PLWH. The absence of financial burdens associated with the prescriptions and access to ART for PLWH correlates with reduced suicide rates among unemployed individuals or those with low household incomes, highlighting a significant policy implication. However, the increased risk of suicide among PLWH who are employed and those with higher household incomes requires future investigation.

When compared with controls, the HR for suicide mortality among PLWH receiving ART was slightly lower than that in all PLWH. This rate is due to free ART prescriptions for PLWH in South Korea [32]. Consequently, PLWH who were not prescribed ART medication are likely to be less willing to seek treatment or have poor compliance with treatment regimens. Additionally, concurrent mental health conditions among PLWH reduced the likelihood of complying with ART [33]. This trend might have marginally increased the risk of suicide among PLWH who do not receive ART.

Our study has some limitations. First, there was a lack of information regarding the CD4 cell count, which is a crucial indicator of HIV activity. This is due to the constraints of the NHIS database. Second, the study did not provide any details regarding the lifestyle factors of PLWH, such as a history of alcohol consumption and tobacco use. Third, although we used PS matching with many variables, residual confounders may have affected the results of this study. Fourth, we used ICD‐10 codes registered by physicians in the NHIS on an individual basis. Thus, data regarding events, such as previous suicide attempts or self‐harm, might have been missed in the diagnostic input. Finally, the generalizability of our findings might be limited because each country has a different medical and social environment and system.

## 5 CONCLUSIONS

This population‐based cohort study in South Korea revealed a higher risk of death due to suicide and other causes among PLWH compared with controls. Furthermore, the risk of death due to suicide was higher than that of other causes among PLWH. In subgroup analyses, the risk of death due to suicide in PLWH was higher than that in controls in those who were male, aged ≥ 40 years) and with CCI ≥2 or underlying cancer. The results of our study will have a significant impact on the development of future strategies aimed at preventing suicide in PLWH.

## COMPETING INTERESTS

All authors have no conflicts of interest regarding this work.

## AUTHORS’ CONTRIBUTIONS

TKO and I‐AS contributed to the study design, analysed the data and drafted the first manuscript. K‐HS, EH and HYP contributed to the data acquisition and critically revised the manuscript. All authors read and approved the final version of the manuscript.

## FUNDING

This research received no specific grant from any funding agency, commercial or not‐for‐profit sectors.

## Supporting information



Supporting Information file 3: Figure S1. Distribution of propensity scores before and after matching.

Supporting Information file 1: Table S1. ICD‐10 codes used to compute Charlson comorbidity index.

Supporting Information file 2: Table S2. Classification of disabilities in South Korea

## Data Availability

The data that support the findings of this study are available from the corresponding author, [I‐AS], upon reasonable request.

## References

[1] Greene WC . A history of AIDS: looking back to see ahead. Eur J Immunol. 2007;37(Suppl 1):S94–SS102. 17972351 10.1002/eji.200737441

[2] Chorba TL , Holman RC , Clarke MJ , Evatt BL . Effects of HIV infection on age and cause of death for persons with hemophilia A in the United States. Am J Hematol. 2001;66(4):229–240. 11279632 10.1002/ajh.1050

[3] HIV/AIDS JUNPo . HIV prevention 2020 road map. Accelerating HIV prevention to reduce new infections by 75. 2017.

[4] Deeks SG , Lewin SR , Ross AL , Ananworanich J , Benkirane M , Cannon P , et al. International AIDS Society global scientific strategy: towards an HIV cure 2016. Nat Med. 2016;22(8):839–850. 27400264 10.1038/nm.4108 PMC5322797

[5] World Health Organization . Suicide worldwide in 2019: global health estimates. 2021.

[6] OECD . OECD health data. 2019. [cited 27 Jul 2021]. Available from: https://data.oecd.org/healthstat/suicide‐rates.htm.

[7] Lee SU , Park JI , Lee S , Oh IH , Choi JM , Oh CM. Changing trends in suicide rates in South Korea from 1993 to 2016: a descriptive study. BMJ Open. 2018;8(9):e023144. 10.1136/bmjopen-2018-023144 PMC6169778 30269071

[8] Du X , Zhang Q , Hao J , Gong X , Liu J , Chen J. Global trends in depression among patients living with HIV: a bibliometric analysis. Front Psychol. 2023;14:1125300. 36968702 10.3389/fpsyg.2023.1125300 PMC10036061

[9] Deshmukh NN , Borkar AM , Deshmukh JS. Depression and its associated factors among people living with HIV/AIDS: can it affect their quality of life? J Fam Med Prim Care. 2017;6(3):549–553. 10.4103/2249-4863.222016 PMC5787953 29417006

[10] Li L , Lee SJ , Thammawijaya P , Jiraphongsa C , Rotheram‐Borus MJ . Stigma, social support, and depression among people living with HIV in Thailand. AIDS Care. 2009;21(8):1007–1013. 20024757 10.1080/09540120802614358 PMC2803757

[11] Armoon B , Fleury MJ , Bayat AH , Fakhri Y , Higgs P , Moghaddam LF , et al. HIV related stigma associated with social support, alcohol use disorders, depression, anxiety, and suicidal ideation among people living with HIV: a systematic review and meta‐analysis. Int J Ment Health Syst. 2022;16(1):17. 35246211 10.1186/s13033-022-00527-w PMC8896327

[12] Lu TH , Chang HJ , Chen LS , Chu MH , Ou NM , Jen I . Changes in causes of death and associated conditions among persons with HIV/AIDS after the introduction of highly active antiretroviral therapy in Taiwan. J Formos Med Assoc. 2006;105(7):604–609. 16877243 10.1016/S0929-6646(09)60158-3

[13] Pelton M , Ciarletta M , Wisnousky H , Lazzara N , Manglani M , Ba DM , et al. Rates and risk factors for suicidal ideation, suicide attempts and suicide deaths in persons with HIV: a systematic review and meta‐analysis. Gen Psychiatr. 2021;34(2):e100247. 33912798 10.1136/gpsych-2020-100247 PMC8042999

[14] Kim C , Jin H , Kang G , Dusing GJ , Chum A. Patterns of follow‐up mental health care after hospitalization for suicide‐related behaviors among older adults in South Korea. J Affect Disord. 2024;350:313–318. 38237869 10.1016/j.jad.2024.01.089

[15] Von Elm E , Altman DG , Egger M , Pocock SJ , Gøtzsche PC , Vandenbroucke JP , et al. The Strengthening the Reporting of Observational Studies in Epidemiology (STROBE) statement: guidelines for reporting observational studies. Lancet. 2007;370(9596):1453–1457. 18064739 10.1016/S0140-6736(07)61602-X

[16] Lee J , Lee JS , Park S‐H , Shin SA , Kim K. Cohort profile: the National Health Insurance Service–National Sample Cohort (NHIS‐NSC), South Korea. Int J Epidemiol. 2017;46(2):e15. 26822938 10.1093/ije/dyv319

[17] Ruzicka DJ , Imai K , Takahashi K , Naito T. Greater burden of chronic comorbidities and co‐medications among people living with HIV versus people without HIV in Japan: a hospital claims database study. J Infect Chemother. 2019;25(2):89–95. 30396821 10.1016/j.jiac.2018.10.006

[18] Fernando SM , Qureshi D , Sood MM , Pugliese M , Talarico R , Myran DT , et al. Suicide and self‐harm in adult survivors of critical illness: population based cohort study. BMJ. 2021;373:n973. 33952509 10.1136/bmj.n973 PMC8097311

[19] Austin PC. An introduction to propensity score methods for reducing the effects of confounding in observational studies. Multivariate Behav Res. 2011;46(3):399–424. 21818162 10.1080/00273171.2011.568786 PMC3144483

[20] Tsai YT , Padmalatha S , Ku HC , Wu YL , Yu T , Chen MH , et al. Suicidality among people living with HIV from 2010 to 2021: a systematic review and a meta‐regression. Psychosom Med. 2022;84(8):924–939. 36162070 10.1097/PSY.0000000000001127 PMC9553271

[21] Ferlatte O , Salway T , Oliffe JL , Trussler T. Stigma and suicide among gay and bisexual men living with HIV. AIDS Care. 2017;29(11):1346–1350. 28278571 10.1080/09540121.2017.1290762

[22] Nielsen A , Azra KK , Kim C , Dusing GJ , Chum A. Is the association between sexual minority status and suicide‐related behaviours modified by rurality? A discrete‐time survival analysis using longitudinal health administrative data. Soc Sci Med. 2023;325:115896. 37084702 10.1016/j.socscimed.2023.115896

[23] Yoo M , Wang JS , Park SJ , Cha JO , Jung Y , Chung YS , et al. Characteristics of recent HIV infection among individuals newly diagnosed as HIV‐positive in South Korea (2008–2015). Sci Rep. 2022;12(1):10515. 35732657 10.1038/s41598-022-13953-0 PMC9217788

[24] Choi SK , Golinkoff J , Lin WY , Hightow‐Weidman L , Muessig K , Bauermeister J . Current and future perspectives of HIV prevention research among young sexual minority men in South Korea. Arch Sex Behav. 2023;52(2):721–732. 36097068 10.1007/s10508-022-02403-7 PMC9466347

[25] Chattun MR , Amdanee N , Zhang X , Yao Z. Suicidality in the geriatric population. Asian J Psychiatr. 2022;75:103213. 35917739 10.1016/j.ajp.2022.103213

[26] Kim G , Lee MA. Age discrimination and suicidal ideation among Korean older adults. Am J Geriatr Psychiatry. 2020;28(7):748–754. 31926841 10.1016/j.jagp.2019.12.002

[27] Autenrieth CS , Beck EJ , Stelzle D , Mallouris C , Mahy M , Ghys P. Global and regional trends of people living with HIV aged 50 and over: estimates and projections for 2000–2020. PLoS One. 2018;13(11):e0207005. 30496302 10.1371/journal.pone.0207005 PMC6264840

[28] Song A , Koh EJ , Lee WY , Chang S , Lim J , Choi M , et al. Suicide risk of chronic diseases and comorbidities: a Korean case‐control study. J Affect Disord. 2024;349:431–437. 38190857 10.1016/j.jad.2024.01.037

[29] Grobman B , Mansur A , Babalola D , Srinivasan AP , Antonio JM , Lu CY . Suicide among cancer patients: current knowledge and directions for observational research. J Clin Med. 2023;12(20):6563. 37892700 10.3390/jcm12206563 PMC10607431

[30] Webel AR , Schexnayder J , Cioe PA , Zuñiga JA. A review of chronic comorbidities in adults living with HIV: state of the science. J Assoc Nurses AIDS Care. 2021;32(3):322–346. 33595986 10.1097/JNC.0000000000000240 PMC8815414

[31] Raschke N , Mohsenpour A , Aschentrup L , Fischer F , Wrona KJ . Socioeconomic factors associated with suicidal behaviors in South Korea: systematic review on the current state of evidence. BMC Public Health. 2022;22(1):129. 35042490 10.1186/s12889-022-12498-1 PMC8765829

[32] Kim J , Lee E , Park B‐J , Bang JH , Lee JY . Adherence to antiretroviral therapy and factors affecting low medication adherence among incident HIV‐infected individuals during 2009–2016: a nationwide study. Sci Rep. 2018;8(1):3133. 29453393 10.1038/s41598-018-21081-x PMC5816616

[33] Mandlate FM , Greene MC , Pereira LF , Gouveia ML , Mari JJ , Cournos F , et al. Association between mental disorders and adherence to antiretroviral treatment in health facilities in two Mozambican provinces in 2018: a cross‐sectional study. BMC Psychiatry. 2023;23(1):274. 37081470 10.1186/s12888-023-04782-0 PMC10116733

