# Supplementary figures and images for: Risk of suicide in people living with HIV: A nationwide, retrospective population‐based cohort study in South Korea
Source: J Int AIDS Soc. 2025 Jun 5;28(6):e26521. doi: 10.1002/jia2.26521 (PMC12141753; doi:10.1002/jia2.26521)

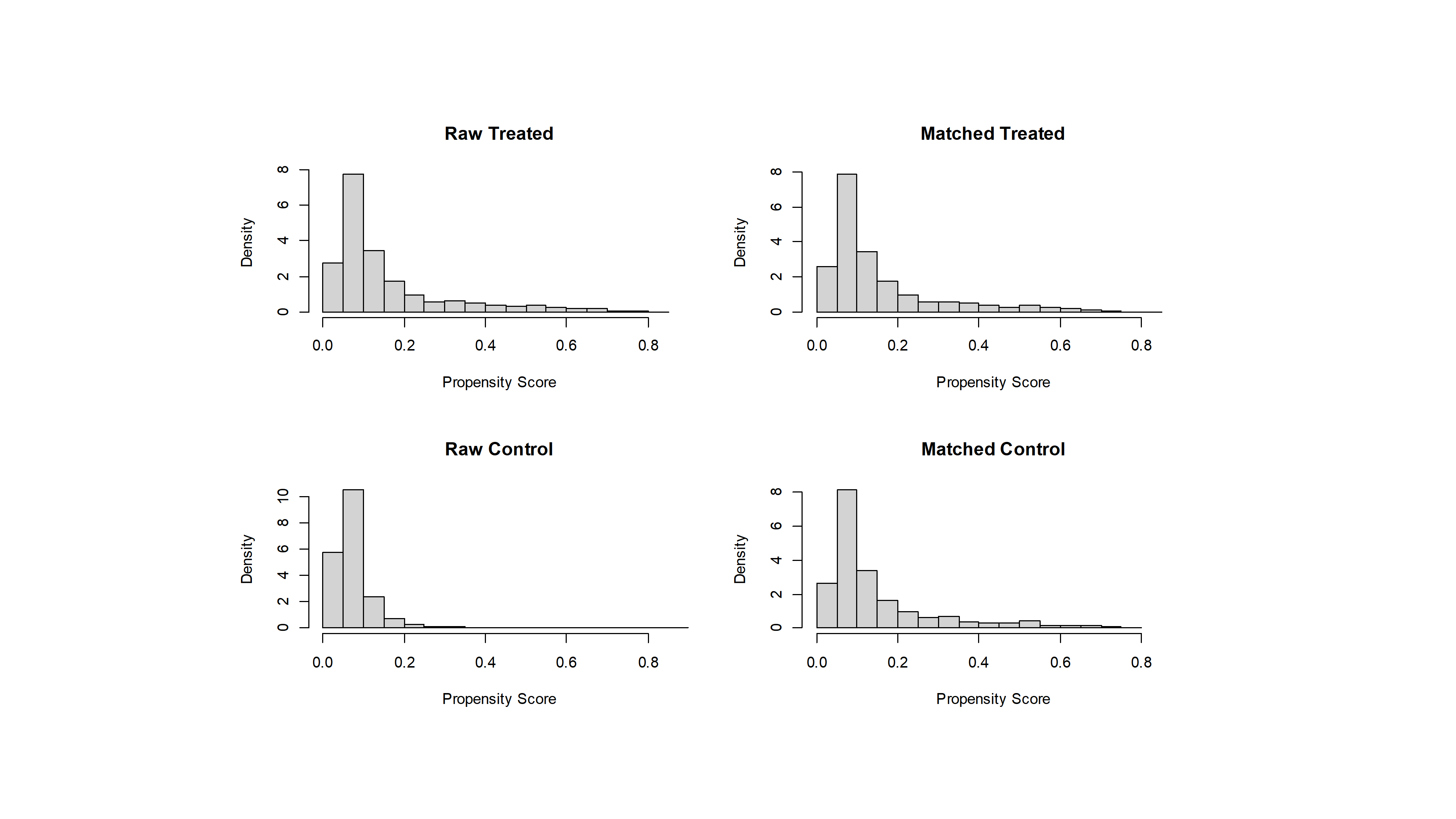

Supplement: Supplementary file 1 — Supporting Information file 3: Figure S1. Distribution of propensity scores before and after matching. [file JIA2-28-e26521-s001.tif]
