# Supplementary material for: Risk of suicide in people living with HIV: A nationwide, retrospective population‐based cohort study in South Korea
Source: J Int AIDS Soc. 2025 Jun 5;28(6):e26521. doi: 10.1002/jia2.26521 (PMC12141753; doi:10.1002/jia2.26521)
Supplement: Supplementary file 3 — Supporting Information file 2: Table S2. Classification of disabilities in South Korea [file JIA2-28-e26521-s002.docx]

Table S2. Classification of disabilities in South Korea

| Type of disability | | Subcategory |
| --- | --- | --- |
|  | Physical disability | Amputation disorder, joint disorder, physical dysfunction, and deformity |
|  | Brain lesion disability | Complex disorders due to brain damage |
|  | Visual disturbance | Blindness, visual impairment |
|  | Hearing disability | Hearing impairment, equilibrium dysfunction |
|  | Speech disability | Language disorder, voice disorder, speech disorder |
|  | Intellectual disorder | IQ is below 70 |
|  | Autism | Autistic disorders such as childhood autism |
|  | Mental disorder | Schizophrenia, schizoaffective disorder, bipolar affective disorder, recurrent depressive disorder |
|  | Renal disorder | Treated by dialysis or have had a kidney transplant |
|  | Heart disorder | Cardiac dysfunction that significantly restricts daily life |
|  | Respiratory disability | Chronic, severe respiratory dysfunction that significantly restricts daily life |
|  | Hepatopathy | Chronic, severe liver function abnormalities that significantly restrict daily life |
|  | Facial disfigurement | Disorders caused by deformities such as abstraction, depression, and thickening of the facial area |
|  | Intestinal and urinary fistulae | Stoma and urostomy that significantly restrict daily life |
|  | Epilepsy | Chronic, severe epilepsy that significantly restricts daily life |
